# The application of social innovation in healthcare: a scoping review

**DOI:** 10.1186/s40249-021-00794-8

**Published:** 2021-03-08

**Authors:** Lindi van Niekerk, Lenore Manderson, Dina Balabanova

**Affiliations:** 1 London School of Hygiene and Tropical Medicine, London, UK; 2 School of Public Health, University of the Witwatersrand, Johannesburg, South Africa; 3 School of Social Sciences, Monash University, Clayton, Australia

**Keywords:** Barriers to care, Healthcare, Social innovation, Systems change

## Abstract

**Background:**

Social innovation has been applied increasingly to achieve social goals, including improved healthcare delivery, despite a lack of conceptual clarity and consensus on its definition. Beyond its tangible artefacts to address societal and structural needs, social innovation can best be understood as innovation in social relations, in power dynamics and in governance transformations, and may include institutional and systems transformations.

**Methods:**

A scoping review was conducted of empirical studies published in the past 10 years, to identify how social innovation in healthcare has been applied, the enablers and barriers affecting its operation, and gaps in the current literature. A number of disciplinary databases were searched between April and June 2020, including Academic Source Complete, CIHAHL, Business Source Complete Psych INFO, PubMed and Global Health. A 10-year publication time frame was selected and articles limited to English text. Studies for final inclusion was based on a pre-defined criteria.

**Results:**

Of the 27 studies included in this review, the majority adopted a case research methodology. Half of these were from authors outside the health sector working in high-income countries (HIC). Social innovation was seen to provide creative solutions to address barriers associated with access and cost of care in both low- and middle-income countries and HIC settings in a variety of disease focus areas. Compared to studies in other disciplines, health researchers applied social innovation mainly from an instrumental and technocratic standpoint to foster greater patient and beneficiary participation in health programmes. No empirical evidence was presented on whether this process leads to empowerment, and social innovation was not presented as transformative. The studies provided practical insights on how implementing social innovation in health systems and practice can be enhanced.

**Conclusions:**

Based on theoretical literature, social innovation has the potential to mobilise institutional and systems change, yet research in health has not yet fully explored this dimension. Thus far, social innovation has been applied to extend population and financial coverage, principles inherent in universal health coverage and central to SDG 3.8. However, limitations exist in conceptualising social innovation and applying its theoretical and multidisciplinary underpinnings in health research.

**Graphic abstract:**

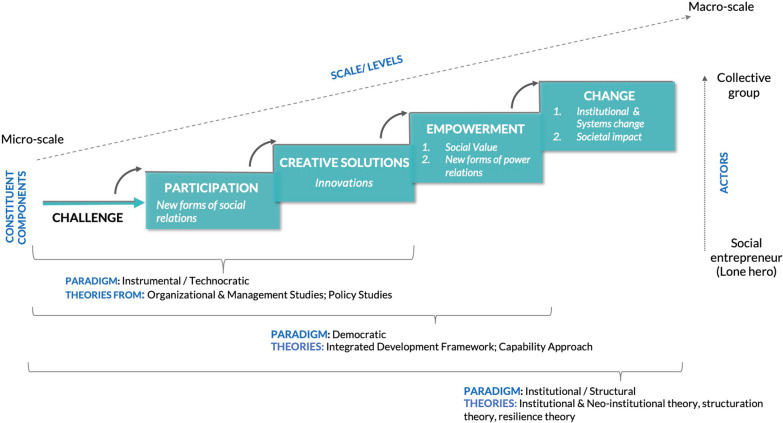

## Background

People cannot operate in a new way unless they can see afresh their real cultural circumstance [1]

The global community has made significant investments in realising health for all people. Yet, despite the ambitious Goal 3 of the Sustainable Development Goals (SDGs), universal health coverage has yet to be experienced by millions of people in high-, middle- and low-income countries [2]. While progress has been made to strengthen health systems, 2020 has been an unprecedented year in which both robust and fragile health systems have encountered significant additional pressures to provide care in the face of the novel coronavirus pandemic, climate-related changes and environmental disasters, economic recession, migration and civil unrest [3–6].

Even prior to the SDGs and most recently the pandemic, social innovation had grown rapidly as an approach to address social challenges across all fields, including in healthcare. The enthusiastic interest in and application of this approach occurred despite a lack of conceptual clarity [7–10]. The hindrance to its wider application, McGowan [11] argues, is that the term ‘social innovation’ has not been employed clearly or consistently.

However, social innovation is regarded as a label for structural change and social reform [12]. From historical accounts, two examples in healthcare are cited as being social innovations: Florence Nightingale’s work, supported by the Irish Sisters of Mercy, in pioneering reform of nursing care [13]; and Cicely Saunders’ creation of what became a global hospice movement for palliative care [14]. Contemporary challenges and the dominant technocratic culture, that often operates at a cost to the human and humane in healthcare systems, services or programme delivery, provide continued impetus for social innovation.

In this article, we consider how social innovation has been applied conceptually in the past 10 years to support the achievement of global health goals, such as universal health coverage. We firstly provide conceptual clarity and framing of the multi-dimensional nature of social innovation, as underpinned by a variety of theories. Secondly, we synthesise the results of a scoping review of peer-reviewed research literature, published in English from 2010 to 2020 on social innovation in health. We conclude by discussing limitations and gaps in the current literature and directions for future research.

## Dimensions of social innovation

### Nature and attributes of social innovation

**Fig. 1:**
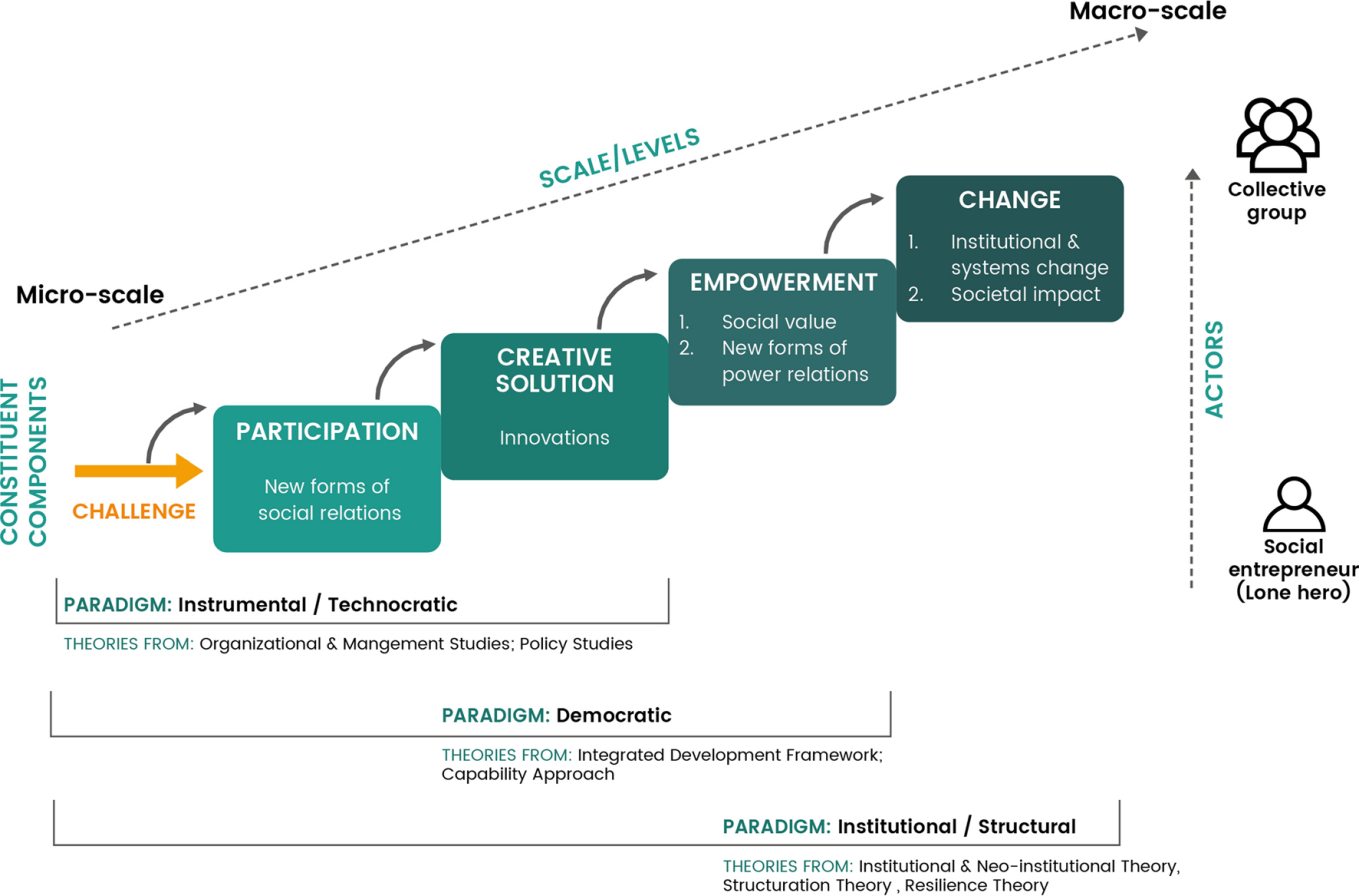
Components, paradigms, theories, scales and actors of social innovation

In 2017, Edwards-Schachter and Wallace [8] conducted a systematic review and identified 252 discrete definitions of social innovations. In this article, we provide a conceptual framing of characteristic aspects of social innovation based on various definitions (Table 1). We seek to highlight the different theoretical applications and paradigms related to social innovation. In Fig. 1, we draw on the work of Ayob et al. [7], and supplement their proposed framing with factors pertaining to understanding social innovation. In the follow text, we briefly discuss each aspect.

**Table 1:** Key social innovation definitions

Theme	Author (year), reference	Definition	Published
Addressing social needs, through new initiatives to improve society	Mumford (2002), [15]	The term social innovation, as used here, refers to the generation and implementation of *new ideas* about how people should organize interpersonal activities, or social interactions, to meet one or more common goals	Creativity Research Journal
	Mulgan (2006), [16]	Social innovation refers to innovative activities and services that are *motivated by the goal of meeting a social need* and that are predominately diffused through organizations whose primary purposes are social	Innovations
	Phillls et al. (2008), [17]	A novel solution to a social problem that is more effective, efficient, sustainable, or just than existing solutions and for which the value created accrues primarily to society as a whole rather than private individuals	Stanford Social Innovation Review
	Pol and Ville (2010), [10]	A *desirable* social innovation is one that in fact (‘in fact’ meaning ‘there is convincing evidence’) improves the macro-quality of life or extends life expectancy	Journal of Socio-economics
	European Commission (2011), [18]	Social Innovation relates to the development of new forms of organization and interactions to respond to social issues (the process dimension). It aims at addressing (the outcome dimension): a. Social demands that are traditionally not addressed by the market or existing institutions and are directed towards vulnerable groups in society. b. Societal challenges in which the boundary between ‘social’ and ‘economic’ blurs, and which are directed towards society as a whole. The need to reform society in the direction of a more participative arena where empowerment and learning are sources and outcomes of well-being	Report: Empowering people and driving change
Forms of participation, relationships and practices	Howaldt et al. (2010), [19]	New forms of *social relations* lead to innovation, which in turn leads to societal impact	
	Neumeier S (2012), [20]	Social innovations as changes of attitudes, behaviour or perceptions of a group of people joined in a network of aligned interests that in relation to the group’s horizon of experiences lead to new and *improved ways of collaborative action* within the group and beyond	European Journal of Rural Sociology
	Cajaiba-Santana (2014), [9]	Social innovations are *new social practices* created from *collective*, intentional, and goal-oriented actions aimed at prompting social change through the *reconfiguration of how social goals* are accomplished	Technological Forecasting & Social Change
Empowering for action	Murray et al. (2010), [21]	Social innovations are new ideas (products, services and models) that simultaneously *meet social needs* and create *new social relationships or collaborations*. In other words, they are innovations that are both good for society and *enhance society’s capacity to act*	Open Book of Social Innovation
	Moulaert et al. (2005 and 2013), [38, 46]	Social innovation as a practice (collective satisfaction of human needs) and a process (changes in social relations, *empowering governance dynamics*) in local development Social innovation refs to changes and agendas, agency and institutions that lead to better inclusion of excluded groups and individuals into various fields of societies at various spatial scales. It is very strongly a matter of process innovation of changes and the dynamics of social relations including power relations’	Urban Studies International Handbook on Social Innovation
Institutional & systems change	Westley et al. (2006, 2010), [22, 23]	Social innovations are products as well as deliberative processes and policies that are *transformative *in their outcome with respect to building greater *social resilience* (Westley, Zimmerman and Patton, 2006) Social innovation is a complex process of introducing new products, processes or programs that profoundly change the basic routines, resource and authority flows, or beliefs of the social system in which the innovation occurs. Such successful social innovations have durability and broad impact	Getting to Maybe (book) The Public Sector Innovation Journal
	van Wijk et al. (2019), [24]	Social innovation for us describes the agentic, relational, situated, and multi- level process to develop, promote, and implement novel solutions to social problems in ways that are directed toward *producing profound change in institutional contexts* (see also Cajaiba-Santana, 2014; Lawrence, Dover, & Gallagher, 2014). We understand this process as embedded and self-reflective, and that it may be coordinated and collaborative, or that it may be the emergent product of accumulation, collective bricolage and muddling through daily work (Garud and Karnøe, 2003; Smets et al. 2012)	Business & Society

### Challenges

The stimulus to social innovation, as for any innovation, is in response to a challenge. By the 1970s scholars had developed an awareness of the limitations of technological innovation and business approaches to effectively meet explicit social needs. Increasingly in the last decade, social innovation has emerged as an alternative to address complex and intransigent societal challenges such as climate change, poverty, the effects of globalisation and inequality, and as a way to produce lasting social change. Social innovation challenges transcend geographic, administrative and political boundaries [9, 25]. For this reason, van Wijk and colleagues argue, challenges best addressed by social innovation have been labelled as ‘wicked problems’ [26], ‘metaproblems’ [27], ‘grand challenges’ [28], or complex challenges with interdependencies across multiple systems and actors [24]. Mulgan [16] highlights the systemic nature of these challenges by noting that existing systems and structures often fail the very people they intend to serve. Others point to the existence of ‘institutional voids’—absent or weak institutional arrangements—in the context of markets and governments that may hinder the participation of communities. The result is that social and economic inequalities emerge or are reinforced [29, 30]. However, Mair argues that these same institutional voids alternatively represent an opportunity for social innovation, allowing new forms of participation by a range of actors with complementary objectives [31].

### Participation

A second distinguishing feature of social innovation, as compared to technological innovation, is its participatory process that promotes social inclusion—reforming existing and promoting inclusive social relationships among individuals, especially those previously neglected from political, cultural or economic engagement [19, 20, 32, 33]. This is often referred to as ‘innovation in social relations’ [15, 34]. It extends beyond the notion of participatory governance, as despite the ability of participatory governance to achieve greater social accountability, it can do so still by focusing only on special interest groups or by limited inclusion [33]. Co-creation, co-production and co-design have become popular mechanisms, used especially by governments, to actively engage citizens in policy and program development [35–37]. Parra [38] connects social innovation with sustainable development, by highlighting how alternative forms of expertise, such as indigenous and citizen knowledge, can result in greater collective learning and knowledge building beyond the technical rationality of scientific protocols.

Four actor groups participating in social innovation are commonly identified: individuals (citizens); social movements; organisations including state and non-state entities (governments, non-governmental organisations, charities, community-based organisations); and new hybrid organisations such as social enterprise [39–41]. Social innovation is unique in terms of cross-boundary or cross-sectoral partnerships at the intersections of business and non-profit sectors. Relationships and trust play an important role in fostering these partnerships [42].

### Creative solutions

Most definitions reference social innovations as creating new ideas or solutions but remain agnostic of the form that this could take being it new products, programs, services, processes, activities, practices or social movements [9, 13, 15, 21, 23, 43]. Yet, social innovations are rarely based on something entirely novel; instead they combine or involve a ‘bricolage’ of two or more existing ideas, theories or products [44]. Diverse theoretical approaches, disciplinary perspectives and even geographic contexts result in different paradigmatic views. One example is the instrumental or technocratic paradigm, originating out of organisational and management studies and public policy from a European context, focused on promoting a neoliberal policy agenda, addressing market failures and reducing public spending [34, 45]. This paradigm regard the most important characteristics of social innovations being ‘more effective, efficient, sustainable or just than existing solutions’, and thus often take the form of social enterprises (or other hybrid organisational models), social finance, corporate social responsibility and public private partnerships [17]. Some scholars have been critical of this paradigm due to its politicised nature. Marques [33] cautions that social innovation can be used as a way of ‘rebranding of political agendas, community development and corporate social responsibility’ by policy makers or academics, without fundamentally altering the goals or outputs. Montgomery [45] warns that the technocratic social innovation solutions could reinforce rather than disrupt top-down vertical power distributions within social relations.

### Empowerment and agency

A second view of social innovation, the democratic paradigm, extends to include components of empowerment and agency [45]. Moulaert [46] regard social innovation as being way to meet human needs by increasing participation levels and empowerment, enabling greater access to resources, and increasing social and political capacities. The quality of participation conceptualised in this view contrasts with that of the technocratic paradigm. While the technocratic paradigm can result in the ‘creative destruction’ of social relations, the democratic paradigm results in the ‘creative transformation of social relations’ [45]. In a case study on the Great Bear Rainforest, Moore and colleagues [47] highlight the role and the redistribution of power between citizens and government in social innovation, that led to governance transformations. Development scholars like Tiwari [48] and Ibrahim [49] have drawn on Sen’s capability approach for human development [50–52] as a way of explaining a bidirectional relationship between agency and social innovation. They argue that through generating agency, social innovations can help achieve new collective capabilities, which can be used by communities to achieve what they value most in life. This work presents a broader view on empowerment, not only as a transfer of power but as the expansion of people’s agency.

### Institutional and systems change

In a subset of definitions, social innovation is presented as institutional change or transformation in complex adaptive systems with authors labelling it the institutional [34], structural or structuration [9, 33] or systemic [22] paradigm. Theoretically it is underpinned by institutional theory, which is regard rules, norms and beliefs as being socially constructed and where micro-level patterns of interaction influence to the creation of macro-level social structures[53]. However, institutional theory does not adequately explain the role of actors in reforming or creating new social systems and structures [9]. Scholars have drawn on neo-institutional and structuration theory to further explore the role of actors as institutional entrepreneurs and their ability to transform the very institutional structures that are meant to constrain action (so called, the paradox of embedded agency) [54–56]. These scholars regard agency as a core catalyst in institutional change which in turn will stimulate transformative change in the social system. In the domain of ecology, scholars have drawn on adaptive cycle heuristic to explain how social innovation generates constant change within social systems by challenging the basic routines, resources, authority flows and beliefs of the social system; so doing social innovation enhances resilience in the system [22, 23, 44, 57]. This approach helps to explain the multi-scalar nature of social innovation—in that micro-level local innovations (within communities and organisations) can cascade up, leading to transformations at larger scales [56].

In summary, social innovation is a multi-dimensional concept that has been studied from different theoretical streams and viewed through different paradigmatic lenses. Beyond regarding social innovations as tangible outputs or solutions, created to address unmet societal needs, social innovations at its core challenges the underlying culture and values of the dominant system. As described above, social innovation also includes innovation in social relations and in power dynamics, leading to governance transformation and changes in internalised (mindsets) as well as externalised (structural) institutions. Social innovation thus holds potential to alter the root issues responsible for systems not delivering their intended objectives to society as a whole.

## Methods

A scoping review was selected as an appropriate method because social innovation has been studied in multiple academic fields such as organisational and management studies, public policy, economics, ecology, urban studies, creativity research and psychology, with each discipline using its own set of research methods. A scoping review assisted us to determine the coverage of the literature on social innovation as pertaining to health, by mapping the available evidence and identifying knowledge gaps or limitations [58, 59]. Three questions were identified to be answered through this review:

- How is social innovation as a concept applied to health, health care or health services?
- What barriers inhibit and what enabling factors support the design and implementation of social innovations in health within the health system or wider context?
- What are the limitations of the current literature on social innovations associated with health systems strengthening?

### Search strategy

Online databases were examined between April–June 2020, including Academic Source Complete, CINAHL, Business Source Complete, Psych INFO, Pub Med and Global Health. Databases were selected for their disciplinary breath. The following search terms were used:

(social innovation [subject heading]; OR “social innovat*” [abstract]; OR “social innovat*” [title]; OR social N1 innovat* [abstract] OR social N1 innovat* [title]) AND health OR healthcare OR health care OR health system OR health services (abstract).

### Inclusion criteria

The inclusion criteria for articles were as follows: (1) published between 2010 and 2020; (2) used the term ‘social innovation’ as a concept and provided a definition; (3) applied social innovation to a dimension of health; (4) described the methods provided; and (5) were available as a full text in English from university databases. A 10-year time frame was selected as it was expected that this period will yield the most significant results, as social innovation research have been on the increase, and also be the most relevant.

### Analytical approach

**Fig. 2:**
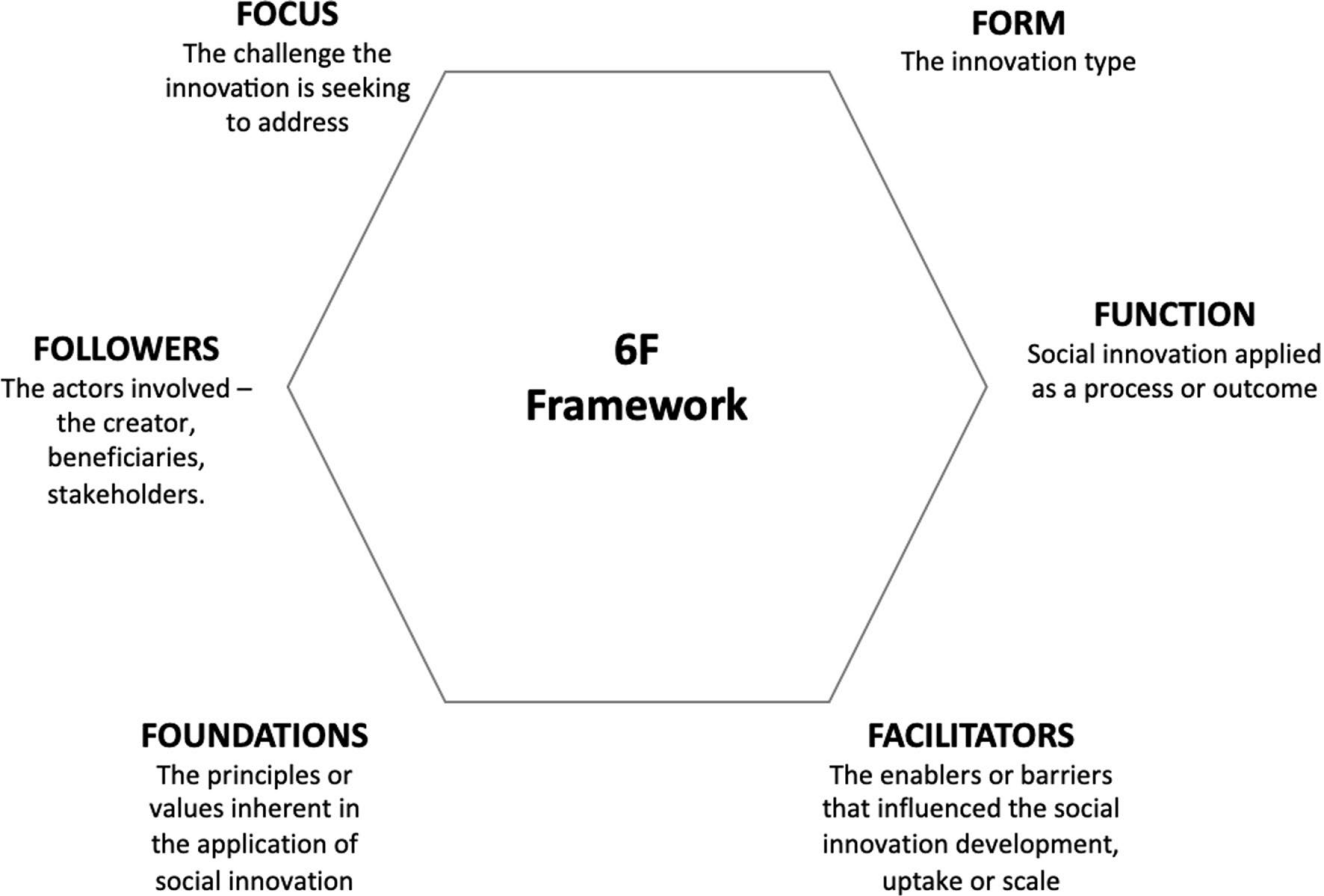
Analytic framework

An analytical framework was developed to assist with analysis, informed by the conceptualisation of the dimensions of social innovation and the framework used by Edwards-Schachter and Wallace [60] (Fig. 1). This framework (Fig. 2) was used to deductively analyse the different aspects of each article included in the review, with NVivo 12 used for the management and coding of material. The framework derived for this study included six areas that contributed to a broad understanding of the literature, as discussed below.

## Results

### Overview of studies included

**Fig. 3:**
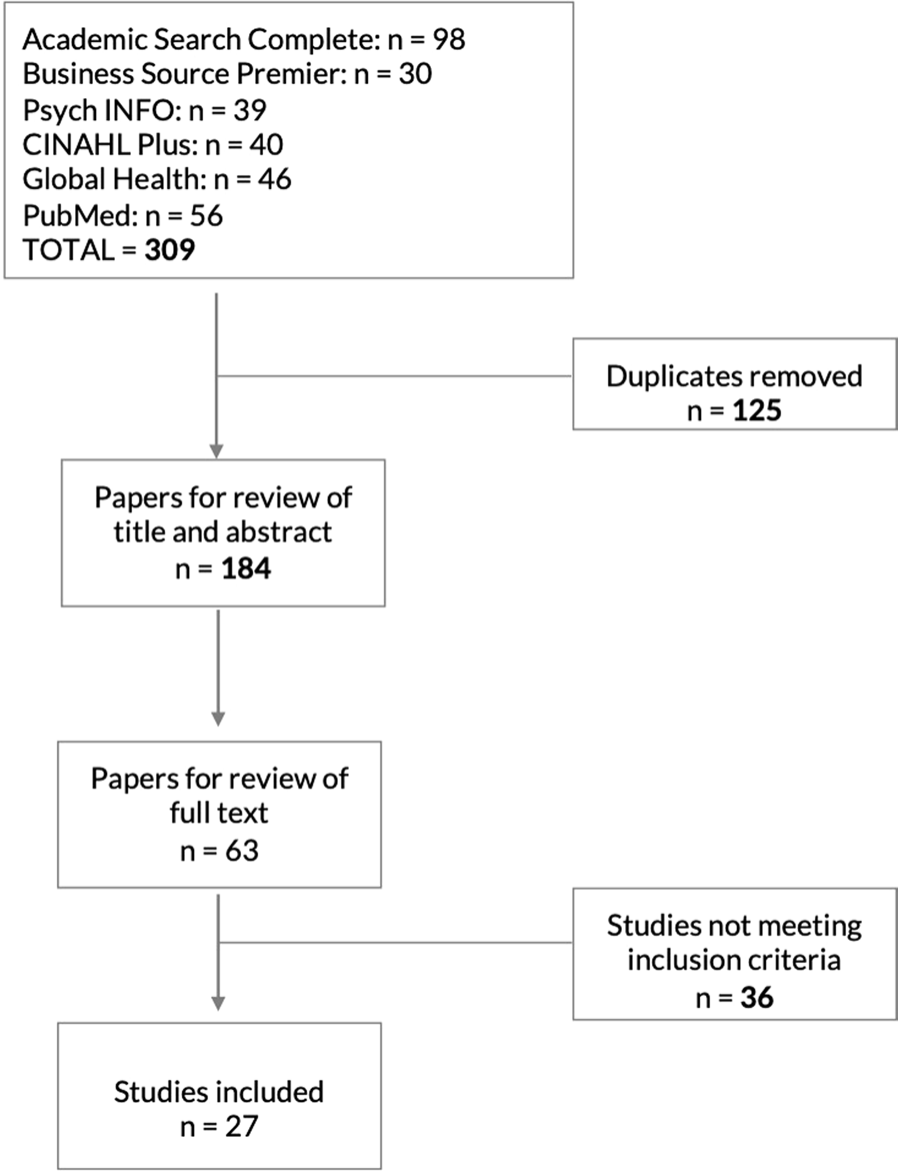
Literature search and review process

A total of 27 studies met the eligibility criteria and were included in the scoping review (Fig. 3). The majority of articles (21/27) were published between 2015 and 2020. Half (14/27) were published in health-specific journals and the remaining half in a range of other disciplines including management and business studies and programme, policy and planning studies, innovation and informatics, and agriculture. The most common methods were case studies (14/27), and scoping, systematic and general literature reviews (4/27). The literature was dominated by research originating from high-income country contexts, particularly in Europe. Nine published studies were conducted in low-income, low-middle income or upper-middle countries (two in Africa; four in Asia; three in Latin America). Low-income country researchers (first author) and institutions were under-represented in the sample, limited to only three representing institutions in Colombia, Uganda and India.

### Focus

Social innovation has been applied to a variety of disease focus areas and to meet public health policy objectives (Table 2). Social innovations in low- and middle-income countries (LMICs), 3/27 studies, focused on infectious diseases, targeting prevention and access to services for malaria, HIV and Chagas disease [61–64]. A second focus of social innovations in LMICs, 9/27 studies, was to achieve equity in access to care and this included women’s health issues and social determinants of health such as poverty, rurality, and infrastructure (basic sanitation) [61, 64, 65]. These focus areas were in line with both national health agendas as well as global agendas as set by the Millennium and Sustainable Development Goals. The literature from high-income countries describes a different application of social innovation in terms of disease focus and public health objectives. Many European countries have adopted social innovation to address welfare state failures, particularly related to the inability of governments to sustain rising health expenditures for ageing populations [66–72]. In this context, social innovations have also been developed in response to policy objectives concerning public participation in health, often as a secondary strategy to move the burden of care from the state to individuals and other actors through social enterprise [71, 73–75]. As this indicates, social innovation is typically applied to address health system failures. Kreitzer et al. [76], for example, explored the Buurtzorg (Neighbourhood Care) Model in the Netherlands, designed to overcome vertical service delivery, low health worker satisfaction, and burdensome bureaucratic processes of care. De Freitas et al. [73] presents a participatory process involving families of patients affected by congenital disorders in the design interventions in areas where health systems responsiveness is poor, and Windrum et al. [77] presents the case of creating a standardised diabetes prevention and management programme based on patient-centred principles. This programme led to the reform of care provision across multiple countries.

**Table 2:** Social innovation challenge focus

Disease focus	Public health objective			
	Health equity (including access & affordability)	Health promotion & prevention	Health system & care-coordination	Expense reduction
Infectious disease	Srinivas et al. (2020), [63]	Castro-Arroyave et al. (2020a), [61] Castro-Arroyave et al. (2020b), [62] Srinivas et al. (2020), [63]		
Non-communicable disease	Mason et al. (2015), [66]	McCarthy et al. (2013), [75] Ruge et al. (2013), [78] Grindell et al. (2017), [79] Windrum et al. (2018), [77]	McCarthy et al. (2013), [75] Henry et al. (2017), [80] Valentine et al. (2017), [81] Windrum et al. (2018), [77]	Dubé et al. (2014), [67]
Maternal, women & child health	Mason et al. (2015), [66] Cheema et al. (2019), [82] Awor et al. (2020), [64]	Castro-Arroyave et al. (2020a), [61]	McCarthy et al. (2013), [75] Dufour et al. (2014), [88] Farmer et al. (2018), [74]	
Ageing population		Ghiga et al. (2020), [83]	McCarthy et al. (2013), [75] Kim HK, et al. (2019), [84]	Currieet al. (2014), [68] De Rosan et al. (2017), [69] Merkel et al. (2018), [70]
Mental health/disability	Mason et al. (2015), [66]	McCarthy et al. (2013), [75]	de Freitas et al. (2017), [73]	
Social determinants of health (poverty, gender, water & sanitation)	Castro-Arroyave et al. (2020a), [61]	Pless et al. (2012), [65]		
No disease focus			Kreitzer et al. (2015), [76] Ballard et al. (2017), [85] Vijay et al. (2018), [86] Cicellin et al. (2019), [72]	Wass et al. (2015), [71] Cicellin et al. (2019), [72]

### Form and function

The classification of social innovations was problematic because of their divergent operational definitions. Two articles provided a proposed typology for social innovations in health. Mason et al. [66] proposed four types of social innovations in health equity: as social movements; services; social enterprises; and digital products. Farmer et al. [74] proposed a typology developed by frontline providers to promote child dental health as: extending existing practices; developing cheaper versions of existing products; adapting existing practices in different contexts or practice spaces; and translating ideas directly from evidence. From these cases studies of specific social innovations, however, the proposed typologies proved too narrow or restrictive as classification structures. The case studies fell into two functional categories, with social innovation treated either as a process or an outcome.

Four studies focused on social innovation as a process. These studies employed participatory mechanisms to support the development of new solutions to local challenges. The goal in all cases was to enhance patient or public participation in health care and enhance social relationships. Collaborative workshops occurred in the form of design sprints, co-design processes and think tank methodologies [73, 74, 81]. All these workshops were led by professional facilitators who were described as being ‘bricoleurs’, providing inspiration to participants, protecting the innovations, and linking them to resources. Srinivas [63], for example, presented a case that used crowdsourcing contests to give men who have sex with men the opportunity to design health promotional material to encourage other men to test for HIV.

Where social innovations were described as an outcome, models included different components (services, products, processes, social movements) and delivery in different settings. Neither single component of the model was particularly unique, but the combination or ‘bricolage’ of these components resulted in innovation. Three types of models were identified: care models (6/27 studies); social network/connection models (3/27 studies); and entrepreneurial models (2/27 studies) (Table 3). These models may or may not have a digital component or a financial component. Innovation in care models involved the re-organisation of care processes, including how services were delivered, often moving facility-based services directly into the community, with the role and scope of providers modified to give more autonomy or allow for task-shifting to non-health professionals [63, 70, 76, 77, 80, 86]. These care models reported positive outcomes on extending access to health services, enhancing affordability and improving effectiveness on disease or wellbeing indicators. The innovative aspect of social network models were the connections and relationships fostered between different actors and sectors [79, 84, 87]. Digital products such as mobile apps or online websites were leveraged to facilitate connections between actors. The outcomes of these models included positive behavioural change, building community social capital, and enhancing women’s participation and roles. The innovation within the entrepreneurial models were mechanisms to reduce costs of services [72, 82], while also improving access to services and creating new employment opportunities.

**Table 3:** Social innovation as an outcome

Theme	Author	Model	Country	Innovator	Location of delivery	Scope and beneficiaries	Components	Reported outcomes
Care models	Kreitzer et al. (2015), [76]	Buurtzog (Neighbourhood Care Model)	Netherlands	A Dutch nurse (Jos de Blok)	Community	630 nursing teams (7188 nurses), 55 000 clients (2013)	Overcoming costly, fragmented home care through: Self-directed, empowered and autonomous nursing teams providing a range of comprehensive services in a relationally oriented way that would achieve patient independence One-cost fee for service with limited managerial staff to keep administrative overhead to a minimum A digital intranet to connect all nurses and perform scheduling, billing, documentation and outcome monitoring	↑ Health worker motivation ↑ Patient outcomes & satisfaction ↓ Fee for service
	Henry et al. (2017), [80]	iMOKO Innovation	New Zealand	A Maori medical doctor (Lance O’ Sullivan)	Community	3800 school-aged children from Maori indigenous group	Overcoming lack of access to care to do place, cultural incongruency and cost of services through: A digital application to support diagnosis and treatment of school-aged children by linking community professionals (eg. teachers) to network of primary care doctors Teachers act as main custodian of school children health	↑ Community ownership over health in line with collectivist cultural values ↓ In indirect costs of accessing care via in person doctor consultation ↑ Affordability of care ↑ Appropriateness of in-person consultations
	Merkel et al. (2018), [70]	Gesundes Kinzigtal (Healthy Kinzigtal)	Germany		Facility		Overcoming fragmented and uncoordinated care through the HK integrated care programme A joint venture between a network of physicians and healthcare management company to extend health services Model supported by two sickness funds and a network 150 partners including allied health services, sports clubs, and self-support groups Outcome-oriented financial approach: profit only made if cost margins of population goes down ie. outcomes improve Provider training in supporting patient self-management and shared decision making Patient accountability through a patient advisory board, satisfaction surveys and patient ombudsman	↑ Patient outcomes ↓ In health expenditure
	Vijay et al. (2018), [86]	Kerala Community Palliative Care	India	Indian medical doctors & volunteers	Community	230 community organizations (85 doctors, 270 nurses 15 000 volunteers, 26 000 social health activist providing care to 70 000 people across 143 villages (2012)	Overcoming access to end of life services and the restrictions of a hospice-based approach: A hub-and spoke model linking community organizations to clinics Non-medical professionals, community volunteers, deliver palliative services Services delivered directly in people’s home	↑ Access to of care ↑ Affordability of care ↑ Awareness of palliative care
	Windrum et al. (2018), [77]	Therapeutic Patient Education	Austria		Facility		Restructuring chronic disease diabetes care according to a patient-centred approach comprised of: Training diabetes educators (different health professionals) and specialist physicians’ postgraduate course Engaging professional associations to set standardised processes for diabetes care and ensuring compliances Including the services as core to the Social Health Insurance fund	↑ Patient knowledge & self-management ↑ Healthy lifestyle behaviour in diabetics
	Srinivas et al. (2020), [63]	Learner Treatment Kit Self-collection for HPV Screening	Malawi Peru	Save the Children & Malawi Ministry of Health University research team	Community	School age children in 58 schools 643 low-income women	Addressing underdiagnosis of malaria in school children due to cost & access to care: Providing a product supply box of malaria diagnostics, treatment and other first aid supplies to schools Training of teachers to administer diagnosis and treatment Addressing cervical HPV screening availability limitations in low-income areas through Leveraging CHWs to provide self-screening kits to women and take kits for diagnostic procedures at health centre Self-testing HPV done by women	↑ Access to of care ↓ School absenteeism
Social-network models	Ruge et al. (2012), [78]	LOMA	Denmark	University research team	Community—Schools		To address obesity among adolescents a multi-strategy approach: Linking schools to local organic food suppliers for local production and procurement Food education for children through linking them to local farmers and combined teacher–pupil cooking classes Shared engagement in meals by teachers and pupils (eating together)	↑ Knowledge of children on food production and nutrition ↑ Scapital between school and local community ↑ Sense of wellbeing through social relationships
	Grindell et al. (2017), [79]	iStep Prototype	United Kingdom	University research team	Community	School-aged children & teachers	To address obesity and limited physical activity in school children through: Pairing up intergenerational teams of school children with teachers or older adults through shared walking challenges A digital pedometer linking to an online platform to measure progress	↑ Physical activity ↑ Social connections
	Kim (2019), [84]	Time Banks	South Korea	American innovator (Edgar S Cahn)—replicated in Korea	Community	950 senior citizens	Addressing the ageing society, high incidence of mental health and suicide in elderly and limited co-ordination between health and social services through: Model that connects people with a need for a service to those who want to serve (creating mutual support network and providing the elderly an opportunity to receive and give services (reciprocity)) Time credits are exchanged for services such as shopping, dog walking, childcare etc	↑ Community solidarity & agency ↑ Individual physical & mental wellbeing ↑ Access to necessary social services ↓ In health-associated costs
Entrepreneurial models	Cheema et al. (2019), [82]	Business-in-a-box	Pakistan	Rural Support Programmes Network (RSPN) in partnership with Population Services International (PSI)	Community	450 women	Addressing low contraception prevalence rate and high unmet need for reproductive health provision through a micro-entrepreneurship approach: Training local women as community resource persons Providing a product kit—a bag with contraceptive, household and hygiene products Establishing a micro-franchise chain to ensure regular product provision	↑ Increase to contraceptives ↑ Female financial independence & empowerment
	Cicellin et al. (2019), [72]	Low cost clinic models	Italy	Centro Medico Santagostino Nuova Citta Medici in Famiglia	Facility		Overcoming service gaps in the national healthcare system for which quality is low or waiting lists are long through different business models that include a social cooperative, a network of low cost clinics These social business models, made possible through: Recruit and engage medical staff at reduced renumeration but with long term financial incentives Different pricing models and a select number of high-value services Operating at economies of scale Cross-subsidization between wealthy and low-income groups or between services generating different profit margins	↑ Affordability of care ↑ Access to of care

### Followers

In the literature, creators of social innovation can operate either as individuals or as collectives, the latter including citizen movements, cross-disciplinary collaborative actor teams and institutions. The characteristics of individual social innovators in health are not well described, but three case studies offer insight into the role of personal experience, hardship or challenge, or of a community playing a significant contribution in the innovator’s work. Among the indigenous Maori population of New Zealand, innovations can often be constrained by culture and place, especially when diverted from acceptable mainstream western approaches [80]. However, social innovators in health used cultural, social and place-based capital to create solutions to serve their own communities [65, 80, 86]. In each case, community trust in the innovation was critical to its success.

The collective creation of social innovation in health (8/27 studies), either in cross-disciplinary actor teams or networks, has received greater attention. Firstly, the social innovation development process is used to overcome the siloed nature of health and to foster greater interdisciplinarity and intersectionality [61, 62, 66, 67, 69, 81, 82, 87]. This is particularly well illustrated in relation to Chagas disease in Guatemala, where innovation in interventions involved collaboration from epidemiology, biology, anthropology, sociology, engineering and architecture, and various funding agencies, international non-governmental organisations, government and universities [61]. The benefit of teams and collective networks is their capacity to move beyond boundaries and draw on collective cognition, capital, and the pooling and complementarity of capabilities [67].

Within these teams, opportunity was created for the participation of non-expert actors. As described in these articles [61, 74, 81], the value of social innovation from a public health policy perspective is the opportunity it affords less powerful actors (patients, families, beneficiaries, community members) to contribute to new health solutions, drawing on experiential knowledge and personal knowledge that can meaningfully contribute to and complement expert or academic knowledge. Applying social innovation as a process in itself leads to new forms of power relations and empowerment. The participation of actors in solution creation in some cases has translated into community action, but little beyond anecdotal evidence is presented in the health literature of sustained intervention success or actor empowerment [61, 73, 74]. Case studies from the management and development literature (3/27 studies) provide more depth and longitudinal evidence to substantiate the extent to which communities can be empowered, ensuring that self-governance and community autonomy of initiatives are achieved. The Kerala Palliative Care model, for example, has scaled far beyond its initial locus of implementation. From 1995 to 2012, 230 community organisations and 26 000 social activists became involved in the delivery of home-based services to 70 000 patients at the end of life [86]. The Graham Vikas social innovation in India also illustrates that the core to its approach is one hundred percent inclusion of members of the community, particularly women’s involvement in all decision-making processes. As a starting point, the program established a representative committee in each village, and a sustainability fund into which community members contributed, according to their means, to co-fund the work. Throughout project implementation, training was delivered on leadership, accounting and other operational procedures to ensure the community can fully manage the initiative independently [65]. Another example, the Business-in-a-Box initiative in Pakistan, illustrates how adopting a micro-entrepreneurship approach to extending access to contraception can empower women to become self-employed income generators while meeting their health needs [82].

In addition to embedding social innovations directly into communities, institutionalised actor networks can work to ensure sustainability. One model which has successfully embedded an initiative across multiple institutional levels is the Therapeutic Patient Education Model for Diabetes [77] in Austria. This case demonstrates the importance of social innovations engaging in institutional and political work with existing professional bodies at local and international levels, while creating new professional bodies to support its translation from research, its diffusion and its sustainability.

In summary, no category of actor is excluded from social innovation, irrespective of his/her background, organisational affiliation or hierarchical level. Across the literature, social innovation is seen as a democratising catalyst for health, enabling broad-based sectoral action, inclusion of marginalised individuals (including women) and providing communities with opportunities for action.

### Values

To examine the principles and values upon which social innovations are based, articles were sub-classified according to the social innovation paradigm to which they ascribed. As illustrated above (Fig. 1), three main paradigms, nested within each other, exist: the instrumental or technocratic paradigm that accounts for social inclusion in the creation of new solutions; the democratic paradigm that accounts for the empowerment of actors through social innovation; and the institutional or structural paradigm that accounts for changes within existing institutions and systems. The majority of articles (16/27) upheld the instrumental or technocratic paradigm in which context social innovation was regarded as a solution to address challenges, and occurred through participatory processes that promoted the social inclusion of different actors. Although encouraging engagement in social innovation, this paradigm does not differ vastly from other approaches to public or patient participation and participatory governance in public health and development. These solutions offer improved ways to ensure greater effectiveness or efficiency, but do not transform relations or structures. These articles originate mainly from Europe, where the approach to social innovation has been influenced by the European Commission’s inclusion of the principle into policy with neoliberal agendas [45].

A second but smaller number of articles (8/27) engage with empowerment. These go beyond giving actors a voice or opportunity to provide input through consultation, and provide them with the opportunity to take control. By building the capacity of marginalised or under-represented actors, they develop an enhanced level of agency and action which suggests a change in power relations taking effect. Many larger-scale social innovation care models had people-centredness as a core organising principle [76, 80, 82]. Models were designed to involve not only the patient or the beneficiary at the health centre, but also health workers. The Buurtzog Neighbourhood Care model, for example, illustrated how, by enhancing patient and provider (nurse) autonomy, better outcomes in care provision were achieved and provider motivation and satisfaction were enhanced [76]. The iMOKO (New Zealand) and Business-in-a-Box (Pakistan) cases both illustrate empowerment of the local community by placing access to healthcare in the hands of trusted community members such as teachers, and by giving women in the community opportunities for income generation [80, 82]. The Time Bank model ascribed dignity and worth to the life of each person, and this highlighted the value of community members as active participants in healthcare: “The first core value of the Time Bank operations is asset, something of value to share with someone else … no one is worthless in the world … everyone is a contributor to society in his or her own way” [84]. Social innovations show how trusted community members such as teachers can play a vital role in promoting health and access to services; how women can play a role in the delivery of health products while being lifted from poverty through income generating opportunities; and how elderly people can be both consumers and providers of services [61, 62, 76, 80, 82, 84, 87].

The third and smallest number of articles (4/27) ascribed and recognised the systemic or structural paradigm of social innovation, and in the research, assessed the changes and dynamics that occurred at an institutional level. The research conducted by Vijay and Monin [86] in India adopted an institutional perspective to examine how certain contexts are more ‘poised’—receptive and ready—for social innovations. They also examined how actors, operating as institutional entrepreneurs, exercised agency to play an important role to increase the readiness of specific context to innovation and overcome the perceived resistance of existing institutions and structures. The Kerala Palliative Care model demonstrated large scale institutional change as it reframed palliative care provision from a medical frame to a social justice frame, with a professional hospice or hospital model replaced by the bottom-up organisation of services delivered primarily by community volunteers. The Therapeutic Patient Education Model for Diabetes revealed that, at the core of this initiative, systems level change was achieved by the institutional work of actors from national professional associations. They worked to embed the model into existing institutions (e.g. health insurance funds), while they created new institutions (new professional bodies) to ensure that new norms, values and practices were embedded at a systems level. Windrum et al. [77] recognised the potential of a model of patient centred care as having the potential of democratising medicine.

Lastly, research conducted by Pless and Appel [65] illustrated how social innovations can transform the norms, values, perceptions and roles within social institutions at community level through several approaches: the complete inclusion of all community members; the establishment of self-governing community structures; the provision of skills building; and service delivery. The project placed community members in the role of clients, so that project staff only acted upon community request. The long-term commitment (> 20 years) of this social innovation ensured that the outcome of an equitable and social society was achievable. This innovation recognised health as an outcome of sustainable development.

### Facilitators and barriers

As a final part of the framework analysis, the facilitators and barriers of social innovations were considered that are relevant at different stages of the social innovation life cycle (Table 4). There were several commonalities across the literature in terms of enablers for idea development and implementation including: creating a safe, protective and facilitated environment; the democratic sharing of knowledge; the importance of timing and context; and implementing self-governance structures to support ongoing implementation and sustainability. Moving beyond the innovation locus to engage more broadly with partners and the existing system influenced innovation transfer, diffusion and scale. Only two studies—Therapeutic Patient Education Model and the Kerala Community Palliative Care model—described the process of institutionalising a social innovation [77, 86]. In both cases, a clear strategic approach was adopted by the innovators and implementers to replace prior institutional logics with new logics. This entailed deep contextual awareness and engagement in different forms of institutional work: advocacy to support movement building; locating the challenge in a moral or social justice frame; engaging existing institutions and creating new ones; and investing in the education of those involved in the innovation, both to attain legitimacy and ensure that standards can be maintained. Both of these social innovations have proven sustainable, and as models, they have been scaled to different settings and countries (Austria and India). Barriers negatively affecting across the various stages of social innovation development included cost considerations and resource constraints, a unreceptive or changing political context, limited evidence of effectiveness and implementer attitudes in terms of low motivation and drive.

**Table 4:** Enablers and barriers

Enablers	Barriers
Stage 1: Idea development & implementation	
A facilitator overseeing the process—guidance, bricolage, linkages with the system [73]	External support—A social innovation process facilitated by professionals would be costly at scale [73]
A protective niche/environment—a safe setting for ideas to be developed and granting participants permission	
Open information sharing between participants and stakeholders across different sectors and disciplines, including involving community or frontline voices [61, 71, 73]	
Timing/Leveraging windows of opportunity—when resources and support is available. [70]	
Context—history of innovation and enterprise in a specific people group, alignment with cultural values, existing organizations, active civic participation [80, 86]	Political context—a changing policy landscape and mandates [88]
Characteristics of the innovator—an insider (from local community, embedded and lived experience), access to different forms of capital (cultural, intellectual, political, social, financial) [65, 80]	Characteristics of implementers—lacking motivation and drive [88]
Community ownership—self-governance structures to place the community (beneficiaries) in charge of the innovation [64, 65]	
Stage 2: Transfer/diffusion/scale	
Alignment with existing regime and structures [74, 77]	Political culture—A lack of willingness of the existing system or government to make allowance for the integration of the innovation or for new actors to play a role [69, 70]
Partnerships with stakeholders & especially policy makers [65, 74]	Resource constraints—limitations in funding [65]
Digital formats e.g. applications, mobile phones, online networks [64, 66, 79]	Limited evidence on social innovation effectiveness and unintended consequences [83, 85]
Stage 3: Institutionalisation	
Political context—encouraging civic engagement and participatory democracy through discussion and deliberation between civil society and state; history of community organizing and social movements; political capacity of government to bring about changes in healthcare [86]	
Communication and advocacy—movement building by engaging a range of organizations to engage in the discussion/spread the message [77, 86]	
Leveraging available infrastructure and competencies ( in contrast to creating new ones)—health facilities, health providers including traditional providers [77, 82, 86]	
Political work—engaging existing institutions e.g., professional associations and forming new ones [77]	
Educating work—developing training for new actors to become involved (medical professionals or volunteers) [77, 86]	
Policing work—through certification of certain actors, quality is enforced and monitored [77]	

## Discussion

Social innovation is a multi-dimensional concept used in relation to innovations in social relations, governance transformation, and social and complex adaptive systems. Actors, as individuals or collectives, play a key role in the social innovation process, especially moving initiatives from a localised level to a macro-level. In this article we sought to critically review the application of social innovation in health care and present the results of a scoping review of peer review research published from 2010 to 2020. In doing this, several research gaps and opportunities for social innovation in health and related research emerged.

The 27 research articles revealed the that social innovation draws on diverse disciplines and fields, with half of the articles arising from fields other than health. Case study research was the main method applied in studying social innovation. As a result, the evidence remains exploratory and descriptive, with weak proof of impact. Most case studies are snapshots of social innovations at specific points in time, without strong theoretical underpinning. No case studies adopted a health systems and policy research perspective. The lack of longitudinal or historic evidence underpinned by theory are barriers to the deeper understanding of the evolutionary process by which social innovation develops, how it is sustained over time through community embeddedness, and how systems change as a result of the adoption and institutionalisation of social innovation. Although research on social innovation in health has increased in recent years, there is still very little research originating from low- and middle-income countries. There is consequently ample opportunity and a need to build stronger evidence on social innovation in health, to deepen the investigation, engage more social scientists, draw on theory from management, organisational and institutional studies, adopt a health systems perspective, and build capacity for this concept and its processes and outcomes in LMICs.

When comparing research conducted and published in health journals with those published in other disciplines, health researchers often adopted a reductionistic view of social innovation, limited to the instrumental and technocratic paradigm of social innovation as a means to an end. Most definitions used to conceptualise social innovation in this literature only addressed the first three dimensions of social innovations: addressing a challenge; adopting a participatory process; and creating solutions. The focus of many of the health solutions presented in this literature was to enhance the effectiveness and efficiency of current health systems. The literature from Europe focused on cost reduction and cost savings to reduce the burden of the state, in line with the neo-liberal political agenda. In this literature, social innovations were described as a variety of disconnected solutions without evidence of how these might act in a coherent and complementary way to achieve systems transformation. This approach appears to re-emphasise the prevailing belief of health systems as mechanistic and compartmentalised, led by technical experts. Social innovation has not been studied through a health systems lens that views systems as social and human institutions [89].

In several studies, the inclusive and participatory process of social innovation has been applied without evidence that led to the empowerment of beneficiaries, patients, frontline workers; social innovation appeared simply as a new buzz word [90]. In line with this, the health literature emphasises the need for facilitators. But cultivating an enabling environment for social innovation does not necessarily require an external, and often costly, facilitator. This current emphasis raises the question whether social innovation is yet another top-down process in health, instead of one that encourages and supports those actors who already demonstrate embedded agency despite constraining institutional structures or settings [55]. For these barriers to be overcome and for social innovation to deliver value, it is imperative to move towards a more democratic and systems paradigm of social innovation. Health researchers would benefit by adopting an interdisciplinary research approach, reviewing and engaging with theories used by other disciplinary scholars, while reflecting on their own expert-driven notions of health.

### Recommendations for policy

Social innovation provides practical insights into how implementation in health systems and practice can be enhanced. It also provides a framework towards understanding systems innovation—the change and transformation of existing systems, beyond mere incremental improvement, or the creation of new systems organised around people’s needs, realities and desires instead of only based on structures solely designed to achieve functional efficiency.

Social innovation supports the development of people-centred systems by suggesting ways to extend the range of actors beyond those traditionally involved in public health programmes. It enhances equity by giving a voice, and thus power, to ideas and solutions, especially those emerging at the grassroots level. By recognising the value inherent in individuals and the knowledge gained from their lived experience, it achieves deeper insight into the structures of power that dictate and limit the roles, capacities and functions of actors and by shifting the power dynamics, new avenues for involvement and participation in health services are created. In addition, social innovation does not seek to provide symptomatic solutions but often addresses the root causes that produce marginalisation, such as addressing community and societal perceptions around the role and participation of women. By design, social innovation initiatives place ‘the last, first’—those with the least experience or least perceived value by society become the creators, drivers and implementers. It invites beneficiaries, frontline providers and community members to be part of the full continuum of implementation, extending them power and agency to become the leaders and ultimately the owners of health interventions and programmes. In this way also addresses the limits of community engagement noted in public health and extends it beyond mere tokenistic consultation [91].

Social innovation’s system’s transforming capacity is further derived from it being inherently interdisciplinary and intersectoral, with boundary-spanning incorporating approaches and practices from different fields and to applied in health care, such as from environmental studies. It thus can be a useful tool for policy makers seeking to enhance holistic socio-developmental policies as espoused in the Sustainable Development Goals, and to solve complex systemic challenges outside sectoral silos.

### Limitations

This scoping review was conducted only on English peer-review literature. Articles in other non-English languages could provide further insights on the concept as applied to health care. A small number of abstracts could not be retrieved via available university access.

## Conclusion

Key in its implementation, social innovation emphasises context. No two contexts are approached in the same way and the nuances and uniqueness are accounted for, so limiting ‘one-size fits all’ models. Case studies illustrate how this has occurred through contextual embedding, adaptation and participation of communities and beneficiaries. Caution should be given however to avoid social innovation becoming a new label for tokenistic participation without a shift in power dynamics across the full spectrum of implementation. Finally, social innovation illustrates the importance of addressing prevailing institutional voids, while holding steadfast the vision of what renewed institutional logics could achieve and providing an inclusive opportunity for all actors to move forward. In this way change occurs slowly, requiring multiple micro-shifts in individuals, communities and health care institutions to ensure sustainability and embedding. To explore the full potential contribution that social innovation offers healthcare, further research is required that adopts an institutional theoretical underpinning and systemic paradigmatic lens.

## Data Availability

The data used for this article is available from the corresponding author on reasonable request.

## Abbreviations

HICs: High income countries
LMICs: Low and middle-income countries
SDGs: Sustainable Development Goals
